# Case Report: Neuroendocrine-marker–negative high-grade neuroendocrine carcinoma mimicking squamous cell carcinoma: an underrecognized diagnostic pitfall

**DOI:** 10.3389/fimmu.2026.1766781

**Published:** 2026-05-08

**Authors:** Wen He, Ying Tang, Yan Li, Jianfeng Zhang, Xuyang You, Qiaozhen Wu

**Affiliations:** 1 Department of Respiratory and Critical Care Medicine, Suzhou Ninth Hospital Affiliated to Soochow University, Suzhou, China; 2 Department of Pathology, Suzhou Ninth Hospital Affiliated to Soochow University, Suzhou, China; 3 Department of Medical Oncology, Suzhou Ninth Hospital Affiliated to Soochow University, Suzhou, China; 4 Department of Nuclear Medicine, Suzhou Ninth Hospital Affiliated to Soochow University, Suzhou, China

**Keywords:** diagnostic pitfall, high-grade neuroendocrine carcinoma, neuroendocrine-marker-negative, POU2F3, YAP1

## Abstract

High-grade neuroendocrine carcinoma (NEC) can be difficult to diagnose, particularly when conventional neuroendocrine (NE) markers are weakly expressed or absent. This challenge is particularly pronounced in NE-low tumors with lineage-defining transcription factor profiles (e.g., POU2F3 and/or YAP1), which may exhibit squamoid (basal-like) morphology and reduced or absent expression of synaptophysin (Syn), chromogranin A (CgA), and CD56, thereby phenotypically overlapping with non-small cell lung cancer (NSCLC) and increasing the risk of misclassification. In this case, needle biopsies from the lung and liver both showed a poorly differentiated carcinoma with squamoid morphology and squamoid/basal-like immunohistochemical features. Syn, CgA, and CD56 were negative in both specimens, leading to an initial diagnosis of poorly differentiated squamous cell carcinoma. However, the subsequent clinical course revealed discordant clinicobiologic features, including rapid progression of the liver metastases, markedly elevated NSE levels, and a high Ki-67 labeling index (60–80%). These discrepancies prompted further molecular evaluation, and 90-gene expression profiling (90-GEP) supported a neuroendocrine lineage assignment. Retrospective immunohistochemistry further demonstrated positivity for POU2F3 and YAP1, whereas INSM1 was negative in both specimens, supporting classification as an NE-marker–negative high-grade NEC with an NE-low NEC/SCLC-like lineage profile. The patient subsequently showed a marked response to etoposide plus carboplatin, consistent with the known chemosensitivity of high-grade NEC/SCLC to platinum-based chemotherapy. This case highlights an underrecognized diagnostic pitfall of NE-marker–negative high-grade NEC with squamoid mimicry. When morphology, immunophenotype, and clinical behavior are discordant, integration of expanded immunohistochemistry (including lineage-defining transcription factors), molecular lineage assays (e.g., GEP), and treatment response may help avoid misdiagnosis and guide appropriate therapy.

## 1 Introduction

High-grade neuroendocrine carcinomas (NECs) are highly aggressive malignancies characterized by rapid clinical progression and overall poor prognosis. Their systemic treatment strategies—particularly platinum–etoposide–based chemotherapy as the therapeutic backbone—differ fundamentally from those of non-small cell lung cancer (NSCLC). Therefore, accurate early classification is crucial for guiding appropriate treatment and improving patient outcomes (1, 2). Misclassification of NEC as NSCLC, particularly as squamous cell carcinoma, may lead to the use of ineffective immunotherapy or NSCLC-directed chemotherapy and thereby delay appropriate treatment.

Although synaptophysin (Syn), chromogranin A (CgA), and CD56 are commonly used immunohistochemical markers of neuroendocrine differentiation, accumulating evidence indicates that approximately 10–20% of small-cell lung cancers (SCLCs) may be negative for both synaptophysin and chromogranin A while retaining CD56 expression in most cases; a smaller subset of NECs (approximately 10%) may be negative for all three markers. In large cell neuroendocrine carcinoma (LCNEC), the expression of NE markers is highly variable, with some tumors showing only focal or low-level expression and others even lacking NE markers entirely (3–5). Small needle biopsy specimens are particularly susceptible to sampling bias because they may capture only non-neuroendocrine–appearing regions of the tumor, thereby further increasing the risk of misdiagnosis. Antigen loss, tumor heterogeneity, and a high Ki-67 index may collectively contribute to the emergence of immunohistochemistry-negative or NE-marker–low NEC, an increasingly recognized diagnostic pitfall in clinical practice (4, 6).

Recent molecular studies have shown that pulmonary small-cell lung cancer (SCLC) can be categorized into four major molecular subtypes based on key transcription factors: achaete-scute homolog 1 (ASCL1), neuronal differentiation factor 1 (NEUROD1), POU class 2 homeobox 3 (POU2F3), and Yes-associated protein 1 (YAP1) (7–9). This classification framework has also increasingly been applied to certain pulmonary high-grade NECs. ASCL1- and NEUROD1-driven tumors generally retain classical neuroendocrine morphology and marker expression, whereas POU2F3- and YAP1-driven tumors typically exhibit NE-low or non-NE lineages with markedly reduced NE marker expression, and may display atypical or partially squamoid/basaloid morphologic features (7). Among these, the POU2F3-driven tuft cell–like subtype has increasingly been recognized as one of the variants most prone to misdiagnosis because it frequently presents with absent neuroendocrine marker expression and partial expression of squamous-lineage markers, resulting in substantial phenotypic overlap with poorly differentiated NSCLC (6, 10). These molecular insights provide a biological explanation for the significant discordance that may arise between morphology and immunophenotype.

When morphology and immunohistochemistry fail to establish a reliable diagnosis, gene-expression profiling (GEP) has emerged as a valuable tool for resolving lineage ambiguity. By analyzing transcriptional signatures rather than protein expression, GEP can reveal occult neuroendocrine differentiation in tumors lacking NE markers. Validation studies—including those assessing the 90-GEP in cancers of unknown primary origin and in gastrointestinal neuroendocrine neoplasms—have demonstrated that GEP can provide reliable lineage information even in cases with non-classical morphology and equivocal immunohistochemistry (11–13).

Here, we report a diagnostically challenging POU2F3/YAP1-associated, NE-marker–negative high-grade NEC. The tumor was initially misdiagnosed as squamous NSCLC on both lung and liver needle biopsies, but the diagnosis was subsequently reversed based on gene-expression profiling and POU2F3/YAP1 immunohistochemistry, followed by a marked clinical response to platinum–etoposide therapy. This case illustrates that when conventional immunohistochemistry is negative, reliance on morphology and conventional immunohistochemistry alone may lead to misdiagnosis, whereas molecular lineage assessment can play a critical role in accurate classification.

## 2 Case presentation

**Figure 1:**
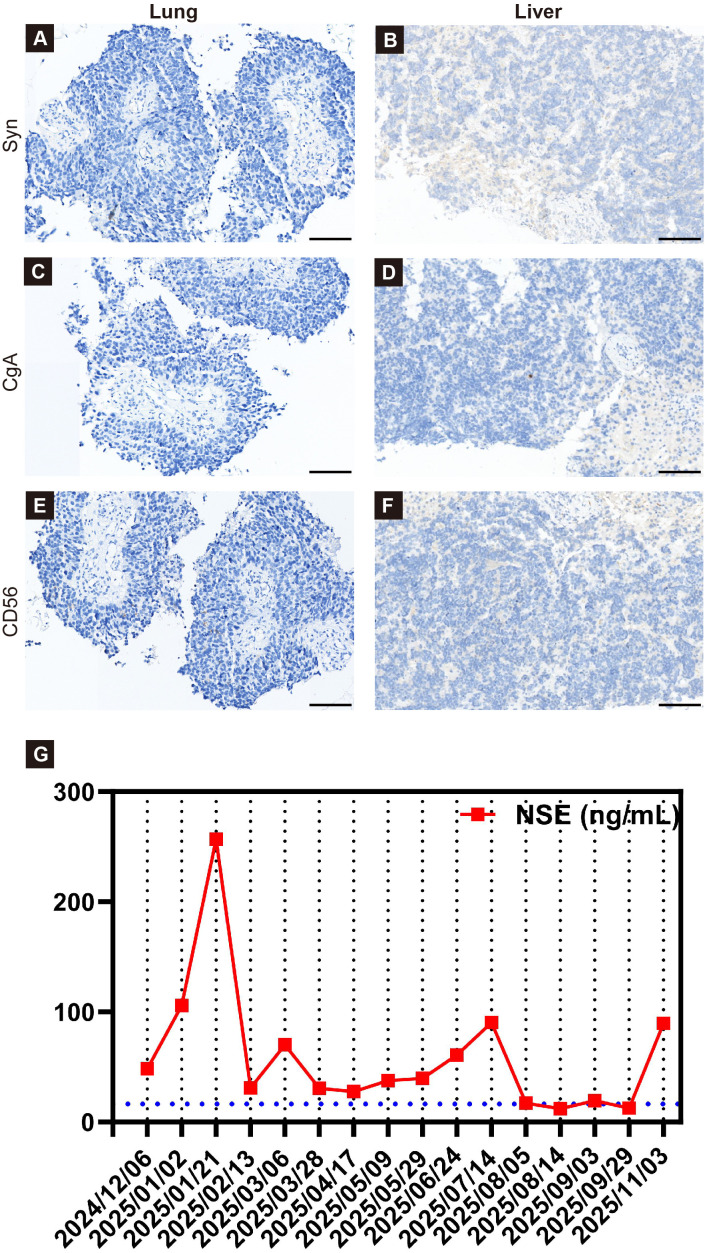
Conventional neuroendocrine marker panel in paired lung and liver biopsy specimens, together with serial serum NSE changes during treatment. The left column shows the lung lesion and the right column shows the liver lesion. **(A, B)** Syn immunohistochemistry is negative in both the lung and liver specimens. **(C, D)** CgA immunohistochemistry is negative in both specimens. **(E, F)** CD56 immunohistochemistry is negative in both the lung and liver specimens. **(G)** Serial serum NSE levels in relation to treatment milestones. Serum NSE concentrations (ng/mL) are plotted over time (red squares connected by a line). The x-axis highlights key time points: 2024/12/06, baseline at hospitalization; 2025/01/21, after two cycles of pembrolizumab plus vinorelbine (NSCLC-directed regimen); 2025/02/13, after switching to etoposide plus carboplatin (SCLC/NEC-directed regimen); 2025/11/03, during terminal deterioration (one week before death on 2025/11/09), with a renewed rise in NSE. The blue dashed horizontal line indicates the upper limit of the normal reference range for serum NSE. Scale bars, 100 μm **(A–F)**.

**Figure 2:**
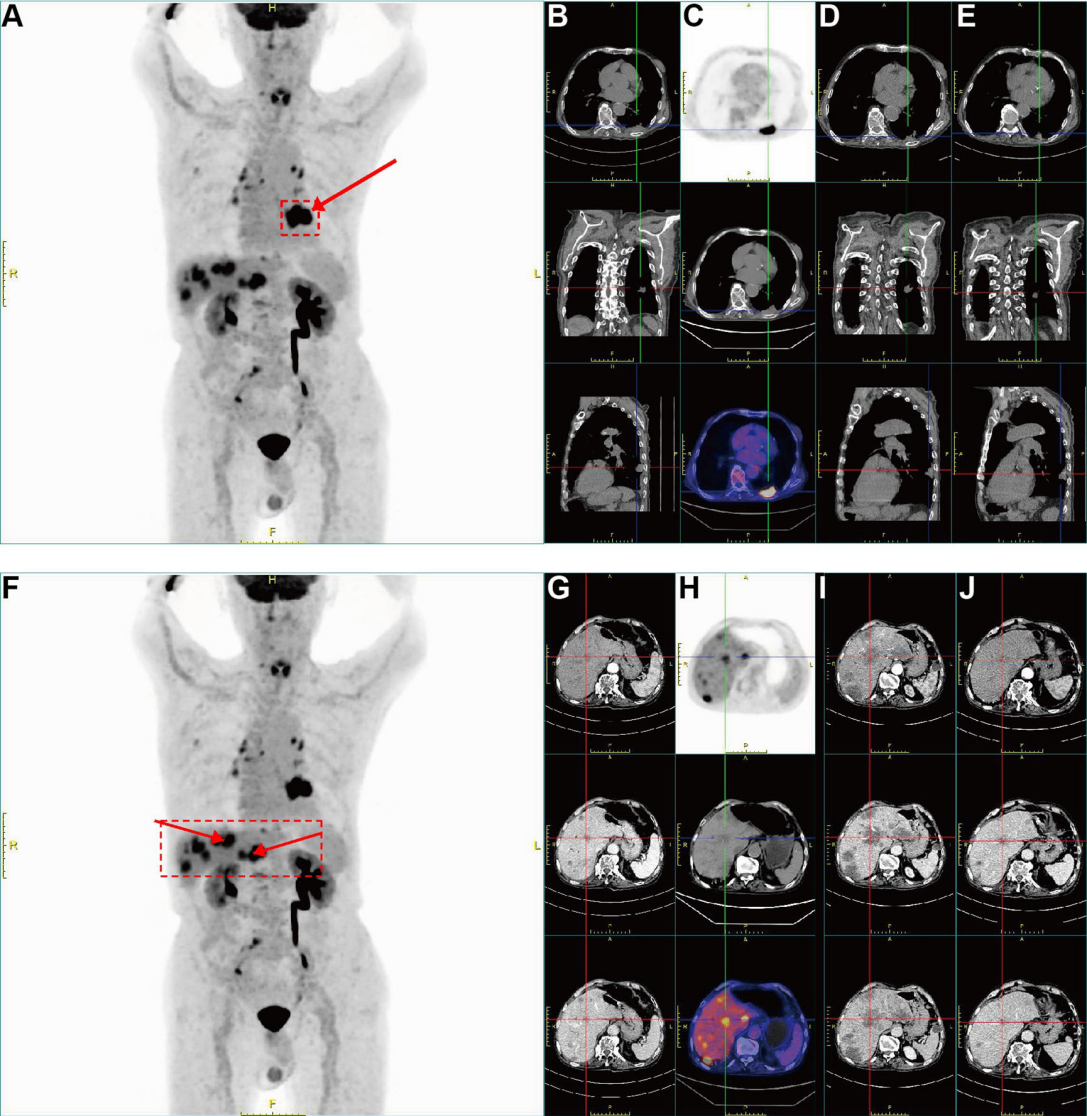
Baseline 18F-FDG PET/CT and serial CT follow-up demonstrating discordant response between the primary lung lesion and hepatic metastases. **(A)** Whole-body maximum-intensity-projection (MIP) 18F-FDG PET image at baseline; arrow indicates the hypermetabolic primary lesion in the left lower lobe (dashed box highlights the lesion-bearing region). **(B)** Axial chest CT at baseline showing a patchy soft-tissue–attenuation lesion in the peripheral left lower lobe with marked emphysema and mildly enlarged mediastinal lymph nodes. **(C)** Corresponding axial PET image confirming prominent FDG uptake in the pulmonary lesion. **(D)** Follow-up chest CT after NSCLC-directed therapy (pembrolizumab plus vinorelbine) showing minimal change, consistent with stable disease, in the pulmonary lesion. **(E)** Follow-up chest CT after switching to etoposide plus carboplatin showing stable-to-slightly decreased pulmonary lesion. **(F)** Whole-body MIP 18F-FDG PET image at baseline highlighting FDG-avid hepatic metastases; arrowheads indicate representative hypermetabolic liver lesions (dashed box outlines the liver-dominant disease region). **(G)** Baseline abdominal CT demonstrating multiple hypodense lesions in both hepatic lobes. **(H)** Corresponding axial PET and fused PET/CT images showing marked FDG hypermetabolism of the hepatic lesions. **(I)** Follow-up abdominal CT after pembrolizumab plus vinorelbine showing rapid progression of liver metastases (increased size/number). **(J)** Follow-up abdominal CT after etoposide plus carboplatin showing marked regression of multiple hepatic metastases, with some lesions nearly resolved.

An 83-year-old man with a smoking history of more than 50 pack-years presented with a 3-day history of left flank and abdominal pain that interfered with daily activities. He denied fever, cough, hemoptysis, and dyspnea. His medical history was notable for schistosomiasis, and there was no family history of lung or liver cancer. Neither germline nor tumor genomic testing was performed, and no relevant psychosocial concerns were identified. On admission, he was afebrile and hemodynamically stable, with a blood pressure of 129/79 mmHg, a heart rate of 76 beats/min, and an SpO_2_ of 98% on room air. Physical examination revealed mild left flank tenderness, without palpable superficial lymphadenopathy, jaundice, or hepatosplenomegaly. Baseline laboratory evaluation showed a WBC count of 6.86×10^9^/L with an absolute neutrophil count (ANC) of 4.92×10^9^/L, hemoglobin of 127 g/L, and platelets of 135×10^9^/L. Liver biochemistry showed AST and ALT of 25 U/L and 10 U/L, respectively, GGT of 79 U/L, total bilirubin of 24.7 μmol/L, and albumin of 42.6 g/L; creatinine was 100 μmol/L. Tumor marker testing showed an NSE level of 48.42 ng/mL and a pro-GRP level of 105.47 ng/mL (**Table 1**, **Figure 1G**). Chest CT (**Figure 2B**) revealed a peripheral lesion in the left lower lobe, together with severe emphysema and mildly enlarged mediastinal lymph nodes. Abdominal CT showed multiple hypodense lesions in both hepatic lobes (**Figure 2G**). ^18F-FDG PET/CT demonstrated intense uptake in the pulmonary and hepatic lesions (**Figures 2A, C, F, H**), raising suspicion for primary lung malignancy with hepatic metastases.

**Table 1:** Serial laboratory parameters across key clinical time points.

Parameter	Baseline	Pre-switch	After EP-C Cycle 2	Terminal deterioration
WBC (×10^9^/L)	6.86	3.6	8.64	8.99
ANC (×10^9^/L)	4.92	2.05	7.12	7.42
Hemoglobin (g/L)	127	104	105	107
Platelets (×10^9^/L)	135	104	159	73
AST (U/L)	25	74	39	197
ALT (U/L)	10	38	19	140
GGT (U/L)	79	334	162	711
Creatinine (μmol/L)	100	108	82	105
ALB (g/L)	42.6	39.8	39.2	30.3
SCC (ng/mL)	1.4	1.1	1	1.1
pro-GRP (pg/mL)	105.47	73.55	64.87	75.16
NSE (ng/mL)	48.42	256.8	70.21	89.51

Laboratory results are summarized at four clinically relevant milestones: baseline (on admission), pre-switch (after NSCLC-directed therapy and immediately before initiation of etoposide–carboplatin), after EP-C cycle 2, and terminal deterioration (during end-stage hepatic failure/cholestasis). Parameters include hematologic indices, liver/renal function tests, and tumor markers to facilitate longitudinal comparison alongside treatment transitions. WBC, white blood cell count; ANC, absolute neutrophil count; AST, aspartate aminotransferase; ALT, alanine aminotransferase; GGT, γ-glutamyl transferase; ALB, albumin; SCC, squamous cell carcinoma antigen; pro-GRP, pro–gastrin-releasing peptide; NSE, neuron-specific enolase; EP-C, etoposide–carboplatin.

**Figure 3:**
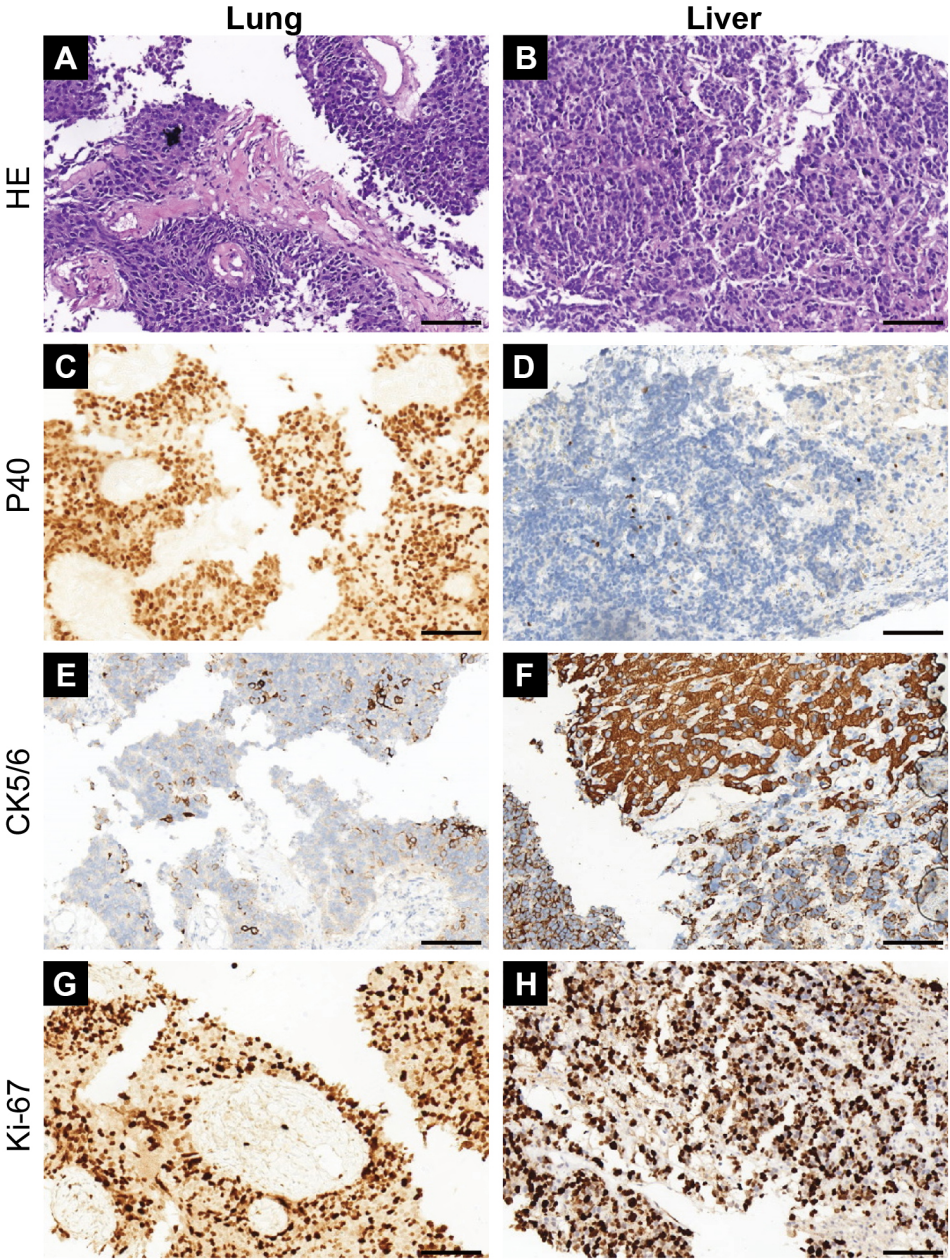
Histopathologic features and immunophenotype of paired lung and liver needle-biopsy specimens. The left column shows the lung lesion and the right column shows the liver lesion. **(A, B)** Hematoxylin and eosin (H&E) staining shows a poorly differentiated high-grade carcinoma composed of densely packed small-to-intermediate tumor cells with scant cytoplasm and hyperchromatic nuclei, with only partial classical neuroendocrine morphologic features; a well-developed salt-and-pepper chromatin pattern and overt nuclear molding are not prominent. **(C, D)** p40 immunohistochemistry shows strong nuclear positivity in a substantial proportion of tumor cells in the lung specimen and only focal weak nuclear staining in the liver specimen. **(E, F)** CK5/6 immunohistochemistry shows focal positivity in the lung specimen and more extensive positivity in the liver specimen. **(G, H)** Ki-67 immunostaining shows a high proliferative index (approximately 67% in the lung specimen and up to 80% in the liver specimen). Scale bars, 100 μm.

Percutaneous biopsy of the lung lesion showed a poorly differentiated carcinoma with squamoid differentiation (**Figure 3A**). On H&E sections, the tumor comprised densely packed small-to-intermediate cells with a high nuclear-to-cytoplasmic ratio, hyperchromatic nuclei, and inconspicuous nucleoli; mitotic activity and apoptotic bodies were observed. The chromatin was focally finely granular but did not show a uniformly classic salt-and-pepper appearance throughout the biopsy specimen. Peripheral palisading was not evident, and nuclear molding was only limited and focal rather than a dominant feature overall. Immunohistochemistry showed strong nuclear p40 positivity in a substantial proportion of tumor cells (**Figure 3C**), focal CK5/6 positivity (**Figure 3E**), and negativity for TTF-1 and Napsin A, supporting a squamoid/basal-like immunophenotype. Syn, CgA, and CD56 were negative in the lung specimen (**Figures 1A, C, E**); the Ki-67 labeling index was 67% (**Figure 3G**). Biopsy of the liver lesion showed a poorly differentiated carcinoma with similar high-grade morphology, composed of solid sheets and nests of small-to-intermediate tumor cells with scant cytoplasm and round-to-oval hyperchromatic nuclei (**Figure 3B**). Similar to the lung biopsy specimen, the chromatin appeared relatively granular but lacked a striking classic salt-and-pepper appearance, and there was no conspicuous peripheral palisading or prominent nuclear molding (**Figure 3B**). The immunohistochemical profile was largely concordant, showing only focal weak nuclear staining for p40 (**Figure 3D**), more extensive CK5/6 positivity (**Figure 3F**), negativity for TTF-1 and Napsin A, and negative staining for Syn, CgA, and CD56 in the liver specimen (**Figures 1B, D, F**), with a Ki-67 labeling index of up to 80% (**Figure 3H**). Accordingly, a preliminary diagnosis of poorly differentiated NSCLC with squamoid differentiation, likely of pulmonary origin with liver metastases, was made.

In December 2024, the patient received pembrolizumab plus vinorelbine based on the initial diagnosis of squamous cell carcinoma. After two cycles, follow-up CT (**Figures 2D, I**) demonstrated minimal change in the primary lung lesion, whereas the hepatic metastases progressed rapidly, with increases in both size and number, consistent with a discordant (mixed) treatment response. During the same period, the serum NSE level increased from 48.42 to 256.8 ng/mL (**Table 1**, **Figure 1G**), accompanied by worsening liver biochemistry, with AST rising from 25 to 74 U/L and GGT from 79 to 334 U/L, as well as declining blood counts, with the WBC count decreasing from 6.86 to 3.6×10^9^/L and the platelet count from 135 to 104×10^9^/L (**Table 1**). Clinically, the patient reported worsening discomfort and reduced activity tolerance. In the context of a high Ki-67 index (60–80%) and the absence of objective benefit from NSCLC-directed therapy, these findings prompted re-evaluation of the initial diagnosis.

Because of this atypical treatment response, a 90-GEP assay was performed on both the lung and liver biopsy specimens. The assay showed high neuroendocrine similarity scores at both sites (79.1% in the lung specimen and 77.8% in the liver specimen), whereas all alternative categories scored low (<6%), thereby supporting a neuroendocrine lineage. Combined with the high Ki-67 index, elevated NSE, and failure of squamous-directed therapy, the diagnosis was revised to NE-marker–negative high-grade NEC, and etoposide plus carboplatin was initiated.

After initiation of etoposide–carboplatin (EP-C), the patient reported gradual symptomatic improvement, with relief of left flank and abdominal pain during the first cycle. His functional status improved, with better appetite and activity tolerance. Consistent with the clinical improvement, the serum NSE level decreased from 256.8 ng/mL before EP-C initiation to 70.21 ng/mL after cycle 2 (**Table 1**, **Figure 1G**). Pro-GRP also showed a downward trend (from 73.55 to 64.87 pg/mL), while SCC remained low and stable (from 1.1 to 1.0 ng/mL). Hematologic parameters recovered after the regimen change: WBC increased from 3.6×10^9^/L to 8.64×10^9^/L, and ANC increased from 2.05×10^9^/L to 7.12×10^9^/L; platelet counts improved from 104×10^9^/L to 159×10^9^/L, whereas hemoglobin remained relatively stable (104–105 g/L). Liver function parameters also improved, with AST/ALT decreasing (74/38 to 39/19 U/L) and GGT declining (from 334 to 162 U/L); albumin remained largely stable (from 39.8 to 39.2 g/L), and no major electrolyte disturbance was observed. After two cycles, follow-up CT (**Figures 2E, J**) demonstrated a significant decrease in multiple hepatic metastases, with some lesions showing near-complete resolution, while the pulmonary lesion remained stable or slightly decreased. Collectively, symptom relief, decreases in NSE and pro-GRP, and radiologic tumor regression after EP-C were consistent with the known chemosensitivity of high-grade NEC/SCLC to platinum–etoposide–based chemotherapy.

After this favorable response, the patient continued neuroendocrine-oriented systemic therapy. Pembrolizumab combined with etoposide and carboplatin was administered from March to June 2025, and re-assessment on May 29, 2025 showed further disease control, with a slight decrease in the pulmonary lesion and continued regression/near-resolution of hepatic metastases. However, CT on July 15, 2025 demonstrated radiologic progression, with enlargement of the lung lesion and increased number and size of hepatic metastases. The regimen was subsequently switched to pembrolizumab plus nab-paclitaxel (July–October 2025). By November 5, 2025, the patient had developed worsening jaundice and liver injury; abdominal CT suggested cirrhotic change with an increased hepatic metastatic burden, whereas the pulmonary lesion remained relatively stable. In the terminal phase, laboratory tests showed marked hepatic injury/cholestasis (AST/ALT 197/140 U/L; GGT 711 U/L) with hypoalbuminemia (30.3 g/L) and thrombocytopenia (73×10^9^/L) (**Table 1**). The patient died on November 9, 2025, after approximately 12 months of follow-up from the initial presentation.

**Figure 4:**
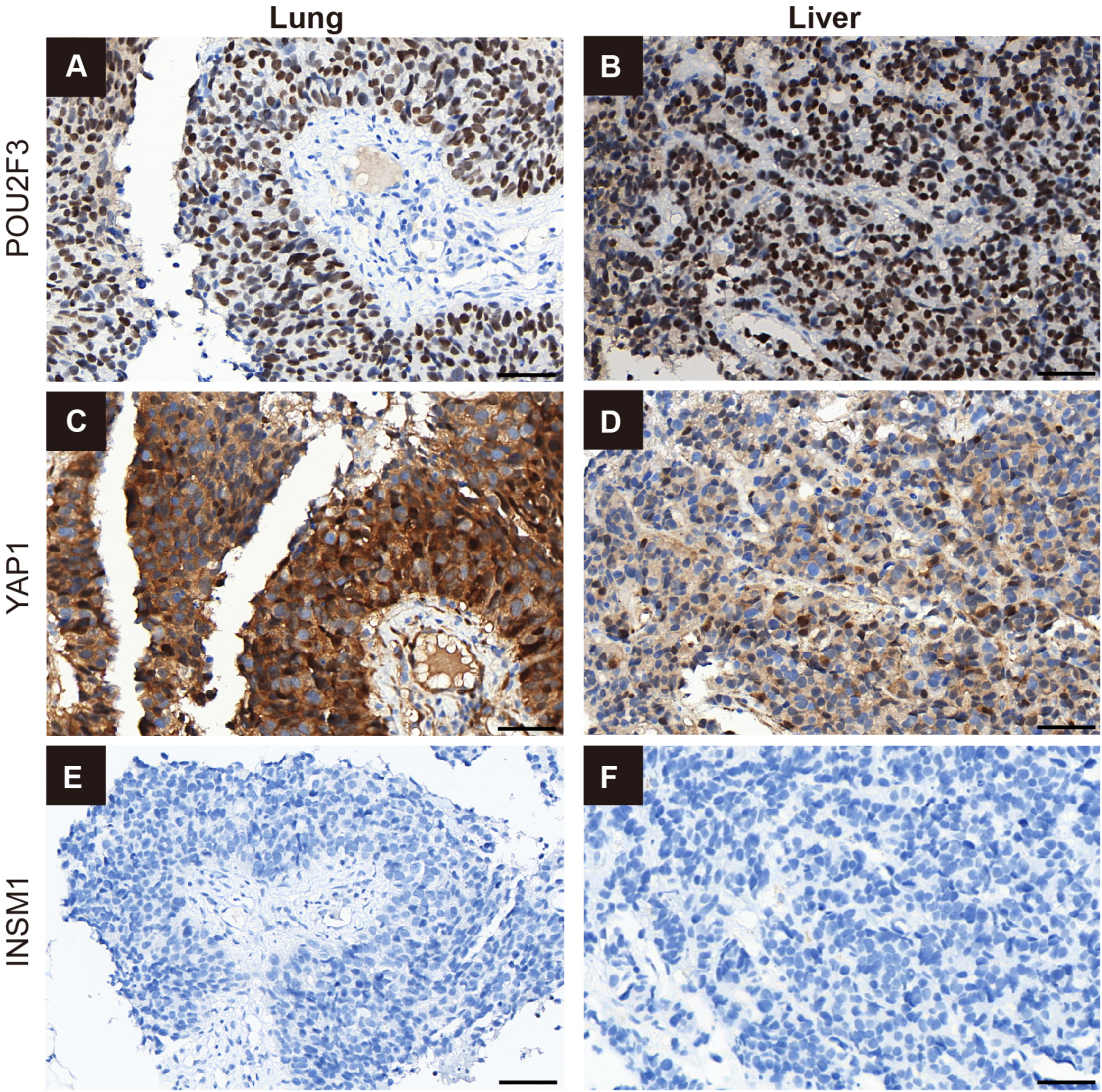
Immunohistochemical demonstration of lineage-associated transcription factors and INSM1 in paired lung and liver biopsy specimens. The left column shows the lung lesion and the right column shows the liver lesion. **(A, B)** POU2F3 immunohistochemistry shows diffuse strong nuclear positivity in both the lung and liver specimens. **(C, D)** YAP1 immunohistochemistry shows tumor-cell positivity, predominantly nuclear with variable cytoplasmic accentuation, with strong diffuse staining in the lung specimen and focal weak-to-moderate positivity in the liver specimen. **(E, F)** INSM1 immunohistochemistry is negative in both the lung and liver specimens. Scale bars, 50 μm.

To further clarify the tumor subtype, additional immunohistochemistry for POU2F3, YAP1, and INSM1 was performed on archived lung and liver biopsy specimens. POU2F3 showed diffuse strong nuclear positivity (3+ in approximately 80–90% of tumor cells) in both the lung (**Figure 4A**) and liver (**Figure 4B**) specimens. YAP1 showed strong diffuse tumor-cell positivity in the lung specimen, with predominantly nuclear staining and variable cytoplasmic accentuation (**Figure 4C**), whereas the liver specimen showed only focal weak-to-moderate positivity (1+–2+ in approximately 10–30% of tumor cells) (**Figure 4D**). INSM1 was negative in both the lung (**Figure 4E**) and liver (**Figure 4F**) specimens. On retrospective review, p40 expression, although appreciable in the lung specimen, did not define a separate morphologically distinct squamous component. These findings make combined SCLC (C-SCLC) less likely and are more consistent with an NE-marker–negative high-grade NEC/SCLC showing POU2F3-high and YAP1-positive features. Overall, this case illustrates an NE-marker–negative high-grade NEC that was initially misdiagnosed as squamous cell carcinoma; its aggressive clinical course, high Ki-67 labeling index, elevated NSE level, and sensitivity to platinum–etoposide chemotherapy supported the final integrated diagnosis.

## 3 Discussion

NEC is a highly aggressive malignancy characterized by marked histologic heterogeneity and often exhibits atypical morphologic and immunohistochemical features in small biopsy specimens, thereby substantially increasing diagnostic difficulty. Traditionally, the diagnosis of NEC has relied on the expression of neuroendocrine markers such as Syn, CgA, and CD56. However, several recent studies have shown that approximately 5–10% of high-grade NECs, particularly pulmonary SCLC and LCNEC, may completely lack expression of classic NE markers on immunohistochemistry, giving rise to what is now recognized as NE-marker–negative or NE-low NEC (3, 4, 14, 15). These tumors often display an undifferentiated basaloid morphology with squamoid differentiation on light microscopy, and their immunophenotype may mimic squamous cell carcinoma, making them highly prone to misdiagnosis as NSCLC (16–18). The present case exemplifies this diagnostic pitfall: both the lung and liver biopsy specimens showed squamoid or undifferentiated morphology; Syn, CgA, and CD56 were negative in both biopsy specimens, collectively creating an immunophenotypic impression of squamous cell carcinoma.

Because morphology and immunophenotype alone are insufficient to resolve lineage in such cases, molecular information becomes essential for reclassification. Transcriptomic analyses have revealed that high-grade NECs, especially those of pulmonary origin, can be categorized into four major molecular lineages driven by ASCL1, NEUROD1, POU2F3, and YAP1 (8). Among these subtypes, ASCL1- and NEUROD1-driven tumors retain classical neuroendocrine differentiation and typically show high expression of Syn, CgA, and INSM1, whereas POU2F3- and YAP1-driven tumors exhibit non-neuroendocrine lineage features and often lack NE marker expression on immunohistochemistry, making them a major source of misdiagnosis in NE-marker–low tumors (7). POU2F3 and YAP1 are key transcription factors associated with the non-classical NE-low lineage program in SCLC (10, 19).

To further refine lineage interpretation, retrospective immunohistochemistry showed diffuse strong nuclear positivity for POU2F3 in both the lung and liver specimens, whereas YAP1 showed strong diffuse positivity in the lung specimen but only focal weak-to-moderate positivity in the liver specimen. In addition, retrospective INSM1 staining was negative in both specimens. Although these findings were highly informative, POU2F3, YAP1, and INSM1 should not be interpreted in isolation. While POU2F3 has relatively high diagnostic utility for NE-low/negative SCLC, with reported sensitivity and specificity of 82.1% and 99.4%, respectively, rare positivity has also been described in pulmonary squamous cell carcinoma (3.13%) (19), and the rate may reach 22% in basaloid squamous cell carcinoma (6). By contrast, YAP1 is less specific, with nuclear positivity reported in approximately 59% of squamous cell carcinomas (20). INSM1, although regarded as a relatively sensitive nuclear marker of neuroendocrine differentiation, is not universally positive in high-grade neuroendocrine carcinomas, and a negative result does not by itself exclude neuroendocrine lineage, particularly in limited biopsy material and in tumors with NE-marker–low features (21). Importantly, prior studies have shown that POU2F3-positive tumors are strongly associated with a neuroendocrine-low phenotype and reduced expression of conventional neuroendocrine markers. In particular, Baine et al. demonstrated that POU2F3-positive SCLC was strongly associated with a neuroendocrine-low phenotype and reduced expression of conventional neuroendocrine markers, including synaptophysin, chromogranin A, CD56, and INSM1, and that POU2F3 was expressed in 75% of SCLC cases with entirely negative or only minimal neuroendocrine marker expression (6). Consistently, Wang et al. identified POU2F3 as a sensitive and specific diagnostic marker for neuroendocrine-low/negative SCLC, further supporting the interpretation that INSM1 negativity in a POU2F3-positive tumor does not contradict neuroendocrine lineage assignment, but rather fits a non-classical NE-low phenotype (19). Therefore, in the present case, the significance of the POU2F3/YAP1/INSM1 profile lies not in any single marker in isolation, but in its interpretation within the overall morphologic, immunophenotypic, clinical, and molecular context. This integrated pattern included negativity for conventional NE markers, a very high Ki-67 labeling index, elevated NSE levels, aggressive clinical behavior, and concordant 90-GEP results. Taken together, these findings suggest that the tumor is most consistent with an NE-low SCLC-P/Y–related lineage or a co-expression/transitional state rather than poorly differentiated squamous cell carcinoma. These findings also argue against C-SCLC because no stable, morphologically and immunophenotypically separable neuroendocrine and non-neuroendocrine components were identified; instead, POU2F3/YAP1 and p40 were largely expressed within the same tumor cell population.

When morphology and immunohistochemistry are both inconclusive, molecular lineage assessment becomes particularly valuable. Recent studies suggest that GEP can provide more stable lineage information when the immunophenotype is ambiguous (11, 22). In our case, 90-GEP on both lung and liver specimens showed high neuroendocrine similarity (79.1% and 77.8%), concordant with rapid progression, a high Ki-67 index, POU2F3/YAP1 positivity, and elevated NSE, thereby supporting an NE-marker–negative high-grade NEC diagnosis and enabling diagnostic revision. When GEP is unavailable, adjunctive immunohistochemical markers should be interpreted cautiously. Although p40 is a useful marker of squamous differentiation, its expression is not entirely specific in the differential diagnosis between squamous cell carcinoma and SCLC. Prior studies have shown that p40 positivity may occur in a subset of SCLCs; Zhang et al. reported a positivity rate of 7.9%, and focal p40 expression has also been described in resected SCLCs. Therefore, p40-positive cells do not necessarily establish squamous lineage and may represent a diagnostic pitfall, particularly in small biopsy specimens (23, 24). In discordant high-grade cases, re-biopsy and an expanded immunohistochemical panel are recommended, including adjunctive neuroendocrine markers such as INSM1 and lineage-defining transcription factors such as POU2F3 and YAP1; ASCL1 and NEUROD1 may be added when appropriate.

The patient’s treatment response provided further functional support for the diagnosis of NE-marker–negative NEC. Standard NSCLC-based therapy (including immunotherapy plus vinorelbine) failed to control the hepatic metastases, which progressed rapidly; in contrast, upon switching to the NEC/SCLC-standard etoposide–platinum regimen, the patient experienced prompt tumor shrinkage, displaying a response pattern consistent with the relative chemosensitivity of high-grade NEC to EP therapy. This distinctive therapeutic sensitivity is a well-recognized biological feature of NEC and, within the molecular subtype framework, may reflect the limited efficacy of NSCLC-directed regimens against NE-low NEC, which nonetheless retains substantial sensitivity to conventional SCLC chemotherapy.

In summary, this case highlights the diagnostic challenge of NE-marker–negative high-grade NEC with squamoid mimicry and the value of integrating clinicopathologic findings with molecular lineage assessment to achieve accurate diagnosis and appropriate therapy.

## 4 Limitations

This report is limited by its single-case nature, and the diagnostic performance of POU2F3/YAP1 immunostaining and 90-GEP in NE-marker–negative high-grade NEC requires validation in larger cohorts. Interpretation of immunohistochemistry and GEP-based lineage assignment may also vary across platforms and laboratories. Comprehensive genomic profiling was not performed. In addition, patient-reported outcomes were collected retrospectively without standardized symptom or quality-of-life instruments, limiting the granularity of clinical assessment.

## 5 Conclusion

NE-marker–negative high-grade NEC with squamoid features may be misclassified as NSCLC on small biopsy specimens. Diagnostic caution is warranted when discordant features arise, including rapid progression, a markedly elevated Ki-67 labeling index, rising NSE levels, or unexpected failure of NSCLC-directed therapy. When available, GEP can provide valuable lineage support; when it is unavailable, repeat biopsy and expanded immunohistochemistry, including INSM1 and lineage-defining transcription factors such as POU2F3 and YAP1, should be considered. In our patient, the response to etoposide–platinum underscored the clinical importance of accurate lineage assignment.

## Data Availability

The original contributions presented in the study are included in the article/supplementary material. Further inquiries can be directed to the corresponding author.
